# *Clostridium sordellii* Pathogenicity Locus Plasmid pCS1-1 Encodes a Novel Clostridial Conjugation Locus

**DOI:** 10.1128/mBio.01761-17

**Published:** 2018-01-16

**Authors:** Callum J. Vidor, Thomas D. Watts, Vicki Adams, Dieter Bulach, Edward Couchman, Julian I. Rood, Neil F. Fairweather, Milena Awad, Dena Lyras

**Affiliations:** a Infection and Immunity Program, Monash Biomedicine Discovery Institute and Department of Microbiology, Monash University, Clayton, Victoria, Australia; b Victorian Bioinformatics Consortium, Monash University, Clayton, Victoria, Australia; c Department of Life Sciences, Centre for Molecular Bacteriology and Infection, Imperial College London, London, United Kingdom; University of Pittsburgh Sch. Med.

**Keywords:** *Clostridium*, *Clostridium sordellii*, conjugation, PaLoc transfer, plasmid

## Abstract

A major virulence factor in *Clostridium sordellii*-mediated infection is the toxin TcsL, which is encoded within a region of the genome called the pathogenicity locus (PaLoc). *C. sordellii* isolates carry the PaLoc on the pCS1 family of plasmids, of which there are four characterized members. Here, we determined the potential mobility of pCS1 plasmids and characterized a fifth unique pCS1 member. Using a derivative of the pCS1-1 plasmid from strain ATCC 9714 which had been marked with the *ermB* erythromycin resistance gene, conjugative transfer into a recipient *C. sordellii* isolate, R28058, was demonstrated. Bioinformatic analysis of pCS1-1 identified a novel conjugation gene cluster defined as the *C. sordellii* transfer (*cst*) locus. Interruption of genes within the *cst* locus resulted in loss of pCS1-1 transfer, which was restored upon complementation in *trans*. These studies provided clear evidence that genes within the *cst* locus are essential for the conjugative transfer of pCS1-1. The *cst* locus is present on all pCS1 subtypes, and homologous loci were identified on toxin-encoding plasmids from *Clostridium perfringens* and *Clostridium botulinum* and also carried within genomes of *Clostridium difficile* isolates, indicating that it is a widespread clostridial conjugation locus. The results of this study have broad implications for the dissemination of toxin genes and, potentially, antibiotic resistance genes among members of a diverse range of clostridial pathogens, providing these microorganisms with a survival advantage within the infected host.

## IMPORTANCE

*C. sordellii* is a bacterial pathogen that causes severe infections in humans and animals, with high mortality rates. While the pathogenesis of *C. sordellii* infections is not well understood, it is known that the toxin TcsL is an important virulence factor. Here, we have shown the ability of a plasmid carrying the *tcsL* gene to undergo conjugative transfer between distantly related strains of *C. sordellii*, which has far-reaching implications for the ability of *C. sordellii* to acquire the capacity to cause disease. Plasmids that carry *tcsL* encode a previously uncharacterized conjugation locus, and individual genes within this locus were shown to be required for conjugative transfer. Furthermore, homologues on toxin plasmids from other clostridial species were identified, indicating that this region represents a novel clostridial conjugation locus. The results of this study have broad implications for the dissemination of virulence genes among members of a diverse range of clostridial pathogens.

## INTRODUCTION

Conjugation is a mechanism of lateral gene transfer by which mobile genetic elements undergo intra- and interspecies horizontal transfer via cell-to-cell contact. Such transfer events can lead to the dissemination of clinically relevant antibiotic resistance- and virulence-related genes. Conjugative plasmids and integrative conjugative elements encode the machinery required for the transfer of a genetic element from a donor cell into a recipient cell (1). The conjugation machinery has two major components. The first is the relaxasome, a protein complex that binds to and processes the DNA for transfer (2). The major component of the relaxasome is the relaxase protein. The relaxase binds to the origin of transfer (*oriT*) of the target DNA, nicks it to produce single-stranded DNA, and facilitates its transfer between donor and recipient cells (1). The second major component is a type IV secretion system (T4SS) that actively pumps the relaxase-bound single-stranded target DNA from the donor cell through a mating channel into the recipient (1).

Conjugation is less well characterized in Gram-positive organisms, and several T4SS components from Gram-negative bacteria are not present (3). The processes occurring at the donor-recipient interface in Gram-positive conjugation systems are still poorly understood; however, cell-to-cell contact is known to be required for conjugative transfer (3, 4), and these systems do contain functional T4SS homologues (3).

In this work, we explored the conjugative transfer of virulence plasmids of the Gram-positive animal and human pathogen *Clostridium sordellii*. *C. sordellii*-mediated infections in animals usually manifest as severe enterotoxemia and can affect cattle (5), sheep (6, 7), and horses (8). Although rare, *C. sordellii*-mediated human infections are often severe, with overall mortality rates reaching 70% (9). The most common human *C. sordellii* diseases appear to be soft tissue infections, which can occur postsurgery or via trauma (9). *C. sordellii* intrauterine infections have also been recorded in women postpartum and postabortion (9–11). These intrauterine infections develop into a toxic shock-like syndrome, and, while such infections are also rare, the mortality rates in these cases are close to 100% (9–12).

Although the pathogenesis of *C. sordellii* infection is still poorly understood, the major virulence factors that lead to severe disease are thought to be two large clostridial toxins (LCTs), TcsH (hemorrhagic toxin) and TcsL (lethal toxin) (13). These toxins are homologues of the better-characterized toxins TcdA (toxin A) and TcdB (toxin B) from *Clostridium difficile*, respectively (14), and TcsL is also similar to Tcnα from *Clostridium novyi* (15) and TpeL from *Clostridium perfringens* (16). The LCTs function by inactivating small GTPases within mammalian cells, leading to an altered cytoskeleton, which results in cell rounding and death (17). The role of TcsL as a crucial virulence factor in *C. sordellii*-mediated disease has been validated by several studies (18–20).

Recent genomic analysis of *C. sordellii* isolates has revealed that TcsH and TcsL are encoded within a pathogenicity locus (PaLoc) related to the *tcdA*- and *tcdB*-encoding PaLoc of *C. difficile* (14). A homologous PaLoc encodes LCT TpeL in *C. perfringens* (21). The genomic context of the LCTs from clostridial species other than *C. sordellii* has largely been characterized, with each of these LCTs either associated with a mobile genetic element (22–25) or able to undergo horizontal gene transfer (26). The *C. difficile* PaLoc in almost all strains is found within a distinct region of the chromosome (27, 28), and while it is not associated with any identifiable mobile genetic elements, conjugative transfer into a nontoxigenic *C. difficile* strain, most likely as a result of random high-frequency recombination transfer of chromosomal DNA, has been demonstrated previously (26). The *tpeL*-associated PaLoc is carried on large conjugative plasmids containing a *tcp* locus from the paradigm *C. perfringens* pCW3 conjugative plasmid (24, 25), and it has been shown that at least one of these *tpeL* plasmids is conjugative (23). The *C. novyi* alpha toxin is carried on a phage genome that has been shown to infect and convert nontoxigenic isolates to a toxigenic phenotype (22, 29).

Until recently, the genomic context of the *C. sordellii* PaLoc was unknown; however, a recent study showed that toxigenic *C. sordellii* strains encoded their PaLoc on members of a related plasmid family, the pCS1 family (30). Four pCS1 family plasmids have been characterized to date (30). pCS1-1 is ~103 kb in size, contains a truncated *tcsH* gene, and is found in the *C. sordellii* type strain, ATCC 9714. pCS1-2 contains a PaLoc related to pCS1-1; however, it is larger (~117 kb) and contains a number of unique regions, while ~106-kb pCS1-3 is the only subtype to contain a full-length *tcsH* gene. Finally, pCS1-4 is smaller (~99.9 kb) and does not contain the PaLoc. In place of this region, there are a number of open reading frames (ORFs) with unknown functions (30). Unlike the state of knowledge of the *C. difficile* PaLoc (26), the *tpeL*-containing plasmids (23), and the *tcn*α-containing phage (22, 29), the capacity of the members of the pCS1 family of plasmids or the *C. sordellii* PaLoc to undergo lateral gene transfer is unknown.

In this work, we sought to functionally characterize the members of the pCS1 family of plasmids and to determine whether they are able to undergo horizontal transfer in *C. sordellii*. Mating experiments showed that a marked version of pCS1-1 from the *C. sordellii* type strain, ATCC 9714, can undergo conjugative transfer into a nontoxigenic *C. sordellii* strain. Bioinformatic analysis of pCS1-1 allowed us to identify a putative novel conjugation locus present on this plasmid. Not only was this locus conserved among all pCS1 family plasmids, but homologous loci were present in other clostridial species, including *Clostridium botulinum*, *C. perfringens*, and *C. difficile*. Finally, insertional inactivation of specific genes within this putative conjugation locus confirmed the functional role that this region plays in the conjugation process.

## RESULTS

### Identification of toxigenic *C. sordellii* isolates and new pCS1 family plasmids.

**FIG 1:**
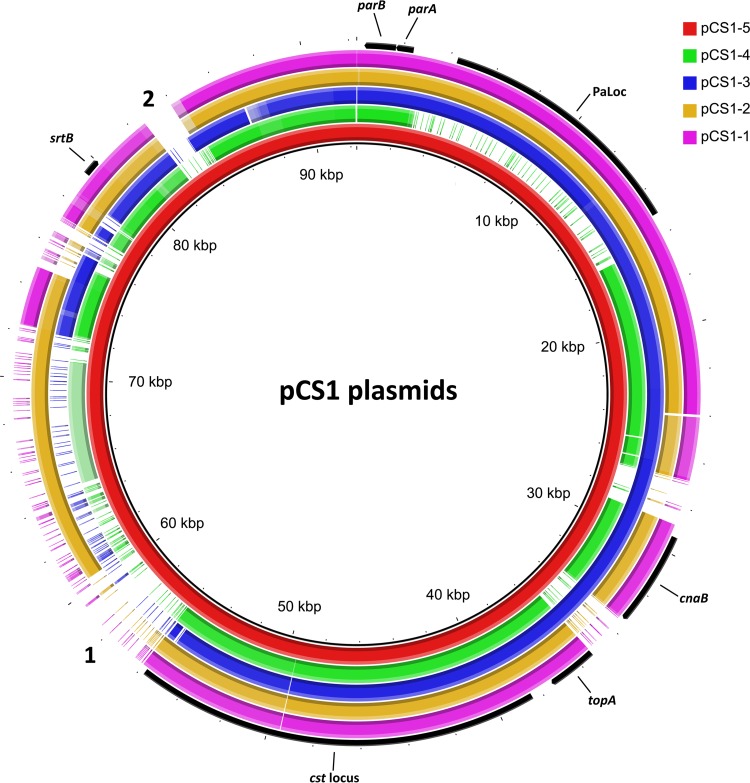
Comparison of pCS1-5 to other members of the pCS1 family. Shown is a visual representation of results of a blastn analysis comparing the pCS1-5 sequence and the other four unique pCS1 plasmid sequences. Each colored ring represents an individual pCS1 member. The innermost ring (colored red) and the coordinates (indicated in kilobases) represent pCS1-5. Plasmids displaying 70% to 100% identity to pCS1-5 at a particular location are represented with a solid block of color on the corresponding ring. Where the level of homology to a particular region is between 50% and 70%, it is represented as a pale block of color. Where identity to a particular region from pCS1-5 is less than 50%, it is represented by a gap on the corresponding ring. Regions present only on pCS1-5 are annotated with a number on the edge of the alignment. Genes and regions of interest from pCS1-1 are annotated on the outermost ring. The figure was produced using BRIG.

To expand on the characterization of the PaLoc located within toxigenic isolates of *C. sordellii*, eight previously uncharacterized *C. sordellii* isolates were screened by PCR for the presence of the *tcsH* and *tcsL* genes (data not shown). One isolate, S0804018, from a case of equine intestinal disease, was *tcsL* positive but *tcsH* negative. The genome of this isolate, along with the genomes of the previously characterized *tcsL*^+^*tcsH*^*+*^ isolates 7543-A and 7508-A, of unknown origin (31), was sequenced. The three isolates were confirmed to encode their LCTs within PaLoc regions identical to those previously characterized (14, 30, 31). With the use of PCR and Sanger sequencing, the contigs containing the PaLoc were closed, confirming that it was carried on a pCS1 family plasmid in each of the strains. The pCS1 plasmids from strains 7543-A (pCS1-6; MG205642) and 7508-A (pCS1-7; MG205641), in addition to being identical to one another, were identical in size (106,013 bp) and had 99% nucleotide identity to pCS1-3 from *C. sordellii* strain JGS6382. The pCS1 plasmid from strain S0804018 (pCS1-5; MG205643) was 92,347 bp in size and is the smallest characterized pCS1 family plasmid identified to date. While regions of pCS1-5 had a high level of nucleotide identity to the established pCS1 members (Fig. 1), a number of ORFs found on the other pCS1 plasmids were absent and two unique regions were present (Fig. 1). On pCS1-5, unique region 1 is predicted to encode hypothetical proteins along with two predicted adenylate/guanylate cyclases (pCS1-5_00054 and pCS1-5_00056). Unique region 2 comprises the majority of a single ORF (pCS1-5_00080), the product of which contains N-terminal DUF5011 domains and C-terminal leucine-rich repeat domains (pfam13306), potentially playing a role as a surface-exposed protein.

### The *C. sordellii* pCS1-1 plasmid can undergo conjugative transfer.

Bacterial matings were performed to determine if a member of the pCS1 plasmid family could undergo conjugative transfer. *C. sordellii* ATCC 9714 derivative DLL5002 was used as a donor as it contained pDLL230, an erythromycin resistance (Erm^r^)-marked version of pCS1-1 (due to TargeTron insertional inactivation of *tcsL*), and was tetracycline sensitive (Tet^s^). Tet^r^ *C. sordellii* isolate R28058 was used as the recipient strain. Erm^r^ Tet^r^ transconjugants were obtained from these matings; however, the level of transfer (55 ± 12.58 CFU/ml [mean ± standard error of the mean {SEM}]; *n* = 3) was low.

To confirm the transfer of pDLL230 from DLL5002 into R28058, six transconjugants from three independent matings were analyzed using phenotypic and PCR screening (data not shown). All transconjugants were deemed to have originated in R28058 and contained the *ermB* gene from the TargeTron procedure. To confirm that the entire pCS1-1 plasmid had transferred into the recipient strain, PCR analysis was performed on 6 transconjugants from two independent matings. ORFs present at various positions around pCS1-1, i.e., ATCC 9714 PCS11_00261 (*topA*), ATCC 9714 PCS11_00521, and ATCC 9714 PCS11_00781, were detected in all transconjugants, suggesting that the whole plasmid had undergone transfer. To confirm this finding, the genome of one transconjugant, DLL5078, was sequenced. Analysis of this genome sequence showed that the chromosome of DLL5078 was indeed of an R28058 background and that it contained the entirety of pDLL230. On the basis of these findings, it was concluded that pCS1-1, and therefore the *C. sordellii* PaLoc, can undergo interstrain horizontal gene transfer.

### The pCS1-1 plasmid is highly stable and contains a ParABS partitioning system.

**FIG 2:**
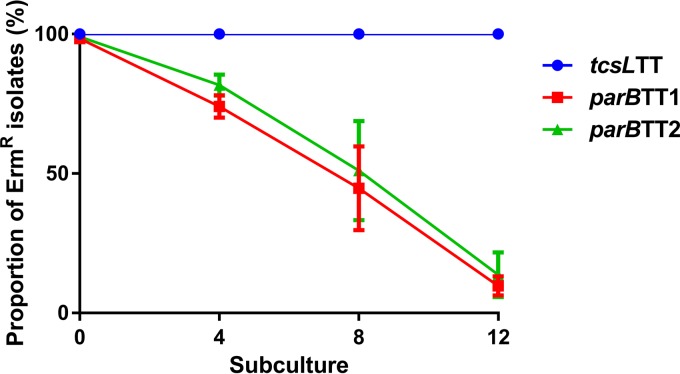
Stability assay demonstrating reduced pCS1-1 stability in the *C. sordellii parB* mutants. The stability of pCS1-1 in *tcsL* TargeTron mutant DLL5002 (*tcsL*TT) and independent pCS1-1 *parB* mutants DLL5138 and DLL5142 (*parB*TT1 and *parB*TT2, respectively) was assessed. Overnight 20-ml BHI broth cultures were inoculated with purified Erm^r^ colonies for all three strains. Each isolate was continuously subcultured 12 times (~120 generations) into fresh 20-ml BHI broth without selection in an attempt to cure the plasmid. Samples were taken from subcultures 0, 4, 8, and 12 and plated out for single colonies. One hundred colonies from each isolate were then patched onto Erm10 BHIS and BHIS plates without selection to determine the percentage of Erm^r^ plasmid-carrying isolates among the members of the population. Means ± standard errors are shown; *n* = 3.

Since the initial matings into the R28058 recipient had a low frequency of pDLL230 transfer, the mating protocol was optimized (see Materials and Methods) and a derivative of ATCC 9714 that could be used as a recipient was constructed. To produce this recipient, it was necessary to cure pCS1-1 from ATCC 9714; however, stability studies performed using both ATCC 9714 and R28058 derivatives containing pDLL230 showed that the plasmid was 100% stable after multiple subcultures (data not shown). All members of the pCS1 family harbor *parA* and *parB* genes (Fig. 1), encoding putative components of an ABS plasmid partitioning system. To determine whether this system was involved in stabilizing the plasmid, the *parB* gene carried on pCS1-1 in ATCC 9714 was insertionally inactivated using a Targetron system (32). The insertional inactivation of *parB* was then confirmed using PCR (data not shown) and Southern hybridization (see Fig. S1 in the supplemental material). A plasmid stability assay was conducted on two independent *parB* mutants and on isogenic *tcsL* mutant derivative DLL5002 (*tcsL*TT), containing an intact *parB* gene. Both *parB* mutants displayed markedly reduced plasmid stability compared to the wild-type plasmid, with the majority (~80% to 95%) of the *parB* mutant populations losing the plasmid over ~120 generations (Fig. 2). This result indicates that the putative partitioning system present on these pCS1 plasmids is required for their stable inheritance.

10.1128/mBio.01761-17.1 FIG S1  Southern hybridization confirmation of TargeTron insertional inactivation mutants of *C. sordellii* ATCC 9714. Genomic DNA was purified and digested with a single restriction enzyme. The Southern blots were probed with DIG-labeled DNA probes specific for (A) the gene of interest; (B) *ermB*, carried within the group II intron; and (C) *catP*, carried on the TargeTron vector backbone. V, HindIII-digested vector control pDLL46; M, DIG-labeled λ-HindIII molecular size markers (sizes given in kilobases); WT, wild-type *C. sordellii* ATCC 9714. (I) Confirmation of independent *parB* mutants. Genomic DNA was digested with XbaI. The TargeTron insertion site is present on a 5,119-bp fragment for the WT, producing an ~6,919-bp fragment in correct mutants. (II) Confirmation of independent *cstD4* mutants. Genomic DNA was digested with XbaI. The TargeTron insertion site is present on a 5,876-bp fragment for the WT, producing an ~7,676-bp fragment in correct mutants. (III) Confirmation of independent *cstB4* mutants. Genomic DNA was digested with AvaII. The TargeTron insertion site is present on a 6,154-bp fragment for the WT, producing an ~7,954-bp fragment in correct mutants. (IV) Confirmation of independent *srtB* mutants. Genomic DNA was digested with PstI. The TargeTron insertion site is present on a 5,150-bp fragment for the WT, producing an ~6,950-bp fragment in correct mutants. Download FIG S1, JPG file, 0.4 MB. Copyright © 2018 Vidor et al. 2018 Vidor et al. This content is distributed under the terms of the Creative Commons Attribution 4.0 International license.

A derivative of ATCC 9714 that had lost pCS1-1 was isolated from one of the colonies from these stability assays, and spontaneous streptomycin (Str; 1,000 μg/ml) and rifampin (Rif; 20 μg/ml) resistance mutants were isolated. One Str^r^ Rif^r^ pCS1-1-free isolate of ATCC 9714 was named DLL5153 and used as the recipient strain for future pCS1-1 matings. The combination of the newly constructed isogenic recipient and optimization of the mating protocol resulted in an ~10-fold to 50-fold increase in the transfer frequency of pCS1-1 to ~1,420 ± 420 CFU/ml (mean ± SEM; *n* = 7).

### A novel conjugation locus present on pCS1 plasmids is related to loci on other clostridial plasmids.

**FIG 3:**
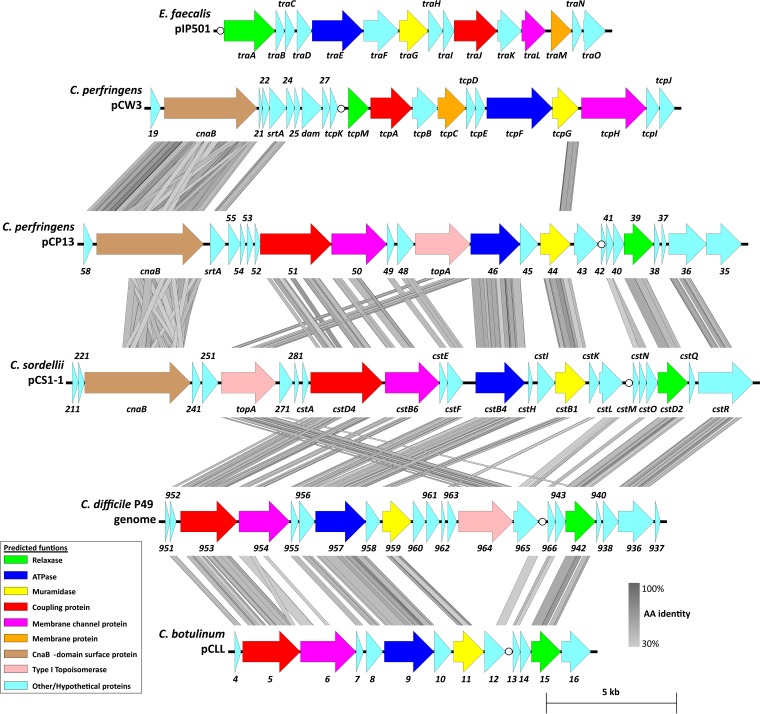
Comparison of the pCS1-1 *cst* locus and surrounding regions with both characterized and putative conjugation loci. Shown is a tblastX alignment of the characterized conjugation locus of broad-host-range plasmid pIP501 (AJ505823, L39769) and *C. perfringens* plasmid pCW3 (DQ366035), along with the putative conjugation locus from *C. perfringens* pCP13 (AP003515), *C. sordellii* pCS1-1 (LN679999), *C. botulinum* pCLL (CP001057), and the *C. difficile* strain P49 draft genome sequence (AVMN01000016). ORFs are colored according to known/predicted function (refer to key). Predicted *oriT* sequences are shown as a small white circle. Regions of amino acid identity between sequences are represented by gray bars. The cutoff value for amino acid identity is 30% across a region of 50 residues, with a maximum E value of 0.001. The higher the level of identity, the darker the gray, as illustrated by the legend. Previously named genes have been labeled as such. ORFs without classical gene names have been assigned a number designation. ORFs within the putative *C. sordellii cst* conjugation locus have been assigned a letter on the basis of the order in which they appear; however, those encoding putative components of a conjugation apparatus have been assigned a suffix based on the corresponding functional homologue from the Vir system of *A. tumefaciens* Ti plasmids (e.g., the VirD4 functional homologue is CstD4). *E. faecalis*, *Enterococcus faecalis*. The figure was produced using EasyFig.

**FIG 4:**
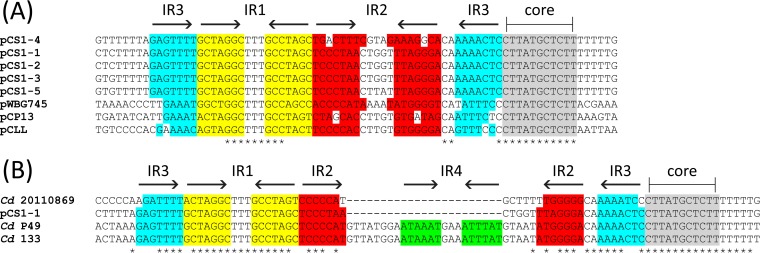
Nucleotide alignments of the predicted *oriT* regions from *cst*-like loci of the clostridia. Multiple-sequence alignments performed using Clustal Omega. The motifs associated with the *oriT* regions of Mob_MG_ relaxase-encoding plasmids are annotated on the alignment with lines/arrows above and highlighting of individual sequences with different colors as follows: gray, highly conserved core (predicted to contain the relaxase nick site [35]); yellow, inverted repeat 1 (IR1); red, IR2; blue, IR3. (A) The predicted *oriT* regions from unique pCS1 family plasmids, as well as those of pCLL and pCP13, were compared to the *oriT* of well-characterized Mob_MG_ relaxase-encoding plasmid pWBG745. (B) The predicted *oriT* regions of *cst*-like loci identified within the genome sequence of *C. difficile* (*Cd*) strains 20110869, P49, and 133 compared with that of pCS1-1. An additional IR, IR4, is annotated above the alignment with inverse arrows and highlighting of individual sequences in green.

To elucidate the mechanism by which pCS1-1 undergoes horizontal gene transfer, bioinformatic analysis of the plasmid was performed. BLAST analysis (33) of several ORFs within an ~17.3-kb region of pCS1-1 identified homologues of the major components of a Gram-positive conjugation system, on the basis of hits in the Conserved Domain Database (34), as well as identifying motifs described in other studies (Table 1). Comparing this region with previously characterized conjugation loci, including the *tra* locus from multispecies Gram-positive plasmid pIP501 and the *tcp* locus from *C. perfringens* plasmid pCW3 (Fig. 3), little similarity was observed. Both the amino acid identities and arrangements of the ORFs differed, indicating that this ~17.3-kb region on pCS1-1 may represent part of a novel conjugation locus, which we have named the *C. sordellii* transfer (*cst*) locus (Fig. 3). ORFs encoding putative conjugation proteins were given a suffix related to their functional homologue within the paradigm Vir system of the *Agrobacterium tumefaciens* Ti plasmids (e.g., for the *virD4* functional homologue, *cstD4*). Any putative genes for which we could not identify a homologue from classical conjugation systems were given a suffix of a letter, from *A* to *R*, ascending in accordance with the order in which they appear 5′ to 3′ on the positive strand of the plasmid. The only well-characterized component of Gram-positive conjugation systems that could not be identified within this locus was a homologue of the VirB8 membrane structural component (3). A putative *oriT* gene was identified upstream of *cstM* comprising a core region (predicted to contain the relaxase nick site [35]) and three inverted repeats (Fig. 4A). This region displays both structural and sequence identity to *oriT* sequences from other Mob_MG_ relaxase-encoding plasmids, including members of the well-characterized pWBG749 family from *Staphylococcus aureus* (35) (Fig. 4A). To determine whether the newly defined *cst* locus is present on all pCS1 family plasmids, a nucleotide BLAST analysis was conducted using the BLAST ring image generator (BRIG) program (36). The alignment demonstrated that the entire *cst* locus is highly conserved among all unique pCS1 plasmids (Fig. 1).

**TABLE 1:** ORFs within an ~17.3-kb region of pCS1-1 predicted to encode major components of a Gram-positive conjugation system^a^

Gene	Predicted function	Bioinformatic evidence
*cstD4*	Coupling protein	C terminus containing pfam12696 domain; mediation of interaction of coupling proteins with cognate relaxase (34); two predicted N-terminal transmembrane helices (3); Walker A and B motifs (61)
*cstB6*	Membrane pore component	Nonspecific/superfamily domain (TIGR02783) found in TrbL (mating pair formation pore-forming protein) of *trb* conjugation locus (34); 5–7 predicted transmembrane domains within the C-terminal half, 698 aa in length, and therefore most similar to class DELTA VirB6-like proteins (3)
*cstB4*	VirB4-like ATPase	Walker A and B motifs (62); multiple domains (pfam12846, TIGR02746, PRK13721) associated with conjugation-related ATPases (34)
*cstB1*	Peptidoglycan hydrolase	Short (22-aa) N-terminal sequence before single transmembrane domain; putative class ALPHA lytic transglycolylase (3); N-terminal nonspecific domains (pfam01464, cd00254) associated with lytic transglycolylases (34); C-terminal catalytic NlpC/P60 domain (34), also present in peptidoglycan hydrolase TcpG from pCW3 conjugation system (63, 64)
*cstD2*	Relaxase	Containing N-terminal catalytic tyrosine motifs and histidine triad motif of Mob_MG_ family of relaxases (clusters within Mob_P_ relaxase family) (35, 65)

a Gene names and predicted functions of their products are shown. The bioinformatic evidence for each ORF’s predicted function is provided together with appropriate references. aa, amino acid.

Comparison of pCS1-1 to plasmids in other species identified a homologous putative conjugation locus present on conjugative botulinum neurotoxin-encoding plasmid pCLL from *C. botulinum* strain Eklund 17B (Fig. 3). Previous studies have shown that regions of pCLL share homology to a region of the *C. perfringens* pCP13 plasmid (37). Alignments of the predicted amino acid sequences from pCS1-1, pCLL, and pCP13 showed a significant degree of homology in both the arrangement of and amino acid sequence identity among both the conjugation-related proteins and hypothetical proteins encoded by these plasmids (Fig. 3; see also Table S1 in the supplemental material).

10.1128/mBio.01761-17.5 TABLE S1  ORFS within and upstream of the *cst* locus. The locus tag corresponding to the published ATCC 9714 pCS1-1 sequence (LN679999), the annotation of the ORF given as part of this study, and the predicted function of the ORF product determined on the basis of homology to the Conserved Domain Database and predicted tertiary structure are indicated. The predicted pCP13 (AP003515), pCLL (CP001057), and *C. difficile* strain P49 (AVMN01000016) homologues to particular ORFs and their homology in amino acid identity determined on the basis of a Clustal Omega multiple-sequence alignment are also provided. Download TABLE S1, PDF file, 0.3 MB. Copyright © 2018 Vidor et al. 2018 Vidor et al. This content is distributed under the terms of the Creative Commons Attribution 4.0 International license.

BLAST analysis performed using predicted proteins of the *cst* locus led to the identification of a *cst-like* locus in *C. difficile* (Fig. 3). This region was identified within contigs of draft genome sequences from *C. difficile* isolates P49 (GenBank accession no. AVMN01000016), 133 (NZ_MBGB01000023), and 20110869 (NZ_MTVT01000059). While not identical, all of the *C. difficile* contigs were of similar sizes, with a significant degree of nucleotide identity observed between them (Fig. S2). Along with the *cst*-like locus, all *C. difficile* contigs encoded plasmid-related genes, including those encoding a ParABS partitioning system and a toxin-antitoxin system. Note that there was a higher level of identity between *cst*-like loci from pCP13 and pCLL and those from pCS1-1 and *C. difficile* sequences, respectively (Table S1). The predicted *cst oriT* regions were also conserved among the clostridial species (Fig. 4); however, the predicted *oriT* of *C. difficile* strains P49 and 133 contains an insertion within IR2, which itself contains a fourth inverted repeat (Fig. 4B). Apart from the presence of homologous *parABS* partitioning genes on pCP13, pCS1, and the *C. difficile* sequences, and with the exception of the conjugation region, no significant identity was seen with any of the remaining plasmids (Fig. S3). This homologous *cst-*like region therefore appears to represent a novel clostridial conjugation locus.

10.1128/mBio.01761-17.2 FIG S2  Nucleotide alignment of the *cst*-like locus containing contigs from draft genome sequences of *C. difficile* strains P49 (AVMN01000016), 133 (NZ_MBGB01000023) and 20110869 (NZ_MTVT01000059). Shown is a visual representation of results of a blastn alignment. Predicted ORFs are represented as blue arrows. Regions of nucleotide identity between sequences are represented by gray bars. The cutoff value for nucleotide similarity was a maximum E value of 0.001 with no minimum length or identity values specified. The higher the level of identity, the darker the gray, as illustrated by the legend. Individual sequences may have been included twice to allow comparisons among all subjects. The figure was produced using EasyFig. Download FIG S2, JPG file, 2.8 MB. Copyright © 2018 Vidor et al. 2018 Vidor et al. This content is distributed under the terms of the Creative Commons Attribution 4.0 International license.

10.1128/mBio.01761-17.3 FIG S3  Nucleotide alignment of the entire sequence of pCS1-1 (LN679999), pCP13 (AP003515), pCLL plasmids (CP001057), and *C. difficile* strain P49 genome sequence contig 15 (AVMN01000016). Shown is a visual representation of results of a blastn alignment. ORFs are colored according to known/predicted function (refer to key). Regions of nucleotide identity between sequences are represented by gray bars. The cutoff value for nucleotide similarity was a maximum E value of 0.001 with no minimum length or identity values specified. The higher the level of identity, the darker the gray, as illustrated by the legend. Individual sequences have been included twice to allow comparisons among all subjects. The figure was produced using EasyFig. Download FIG S3, JPG file, 2.8 MB. Copyright © 2018 Vidor et al. 2018 Vidor et al. This content is distributed under the terms of the Creative Commons Attribution 4.0 International license.

### Insertional inactivation of key genes within the *cst* locus abrogates conjugative transfer.

To determine if genes within the *cst* region of pCS1-1 are responsible for conjugative transfer, mutagenesis of specific genes was performed. The *cstD4* gene (predicted to encode a coupling protein) and the *cstB4* gene (predicted to encode a conjugation-associated ATPase) were individually insertionally inactivated and the *cstD4* and *cstB4* mutants confirmed using PCR (data not shown) and Southern hybridization (Fig. S1). We also wished to examine the role of a putative sortase enzyme, encoded by the *srtB* gene carried on each member of the pCS1 family plasmids (Fig. 1), in conjugative transfer. Independent *srtB* insertional inactivation mutants were constructed and were confirmed by PCR (data not shown) and Southern hybridization (Fig. S1).

**FIG 5:**
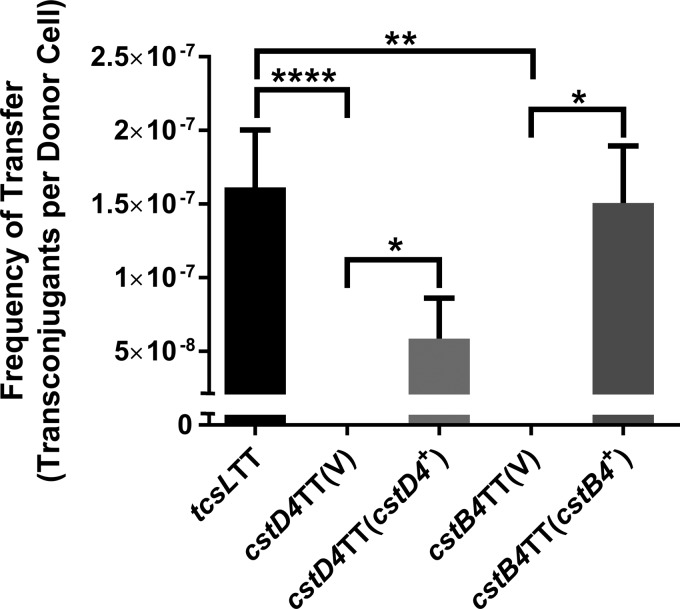
Analysis of pCS1-1 conjugative transfer of insertionally inactivated mutants of putative conjugation genes. Isogenic donor strains of each respective mutant are shown on the *x* axis. Wild-type transfer frequencies are illustrated for the *tcsL* TargeTron mutant “*tcsL*TT.” Shown are conjugation frequencies of *cstD4* and *cstB4* mutants and the corresponding complemented strains. The transfer frequency is expressed as the number of transconjugants per donor cell obtained from each mating. Means ± standard errors are shown; *n* ≥ 4. (V), strain containing vector plasmid pRPF185. Statistical analysis was carried out using a Mann-Whitney *U* test. *, *P* ≤ 0.05; **, *P* ≤ 0.01; ****, *P* ≤ 0.0001.

Mating assays were conducted on the panel of mutants, and transfer frequencies were compared to those of conjugative pCS1-1 derivative pDLL230 from DLL5002 (*tcsL*TT). While the average plasmid transfer efficiencies of independent *srtB* mutants appeared slightly lower than that of the wild-type strain (Fig. S4), variations in transfer frequencies between replicates prevented confirmation of the role of SrtB in conjugation. However, as determined on the basis of these data, and under the conditions tested, SrtB is not essential to the conjugative transfer of pCS1-1. Both the *cstD4* and *cstB4* mutants (carrying the control vector pRPF185) were nonconjugative, with no Erm^r^ transconjugants detected for either mutant (see Materials and Methods for detection limits) (Fig. 5). To confirm the role of *cstD4* and *cstB4* in the conjugative transfer of pCS1-1, the mutants were complemented in *trans* with the wild-type genes, including their ribosome binding site (RBS) cloned into pRPF185. The transfer efficiency was restored in the complemented strains (Fig. 5), albeit not to wild-type levels for the complemented *cstD4* mutant. These results indicated that the products of *cstD4* and *cstB4* were required for pCS1-1 conjugative transfer and confirmed that the *cst* locus represents a novel clostridial conjugation region.

10.1128/mBio.01761-17.4 FIG S4  Analysis of pCS1-1 conjugative transfer of independent insertionally inactivated mutants of *srtB*. Isogenic donor strains of each respective mutant are indicated on the *x* axis. Wild-type transfer frequencies are illustrated by the *tcsL* TargeTron mutant “*tcsL*TT.” Transfer frequency is expressed as the number of transconjugants per donor cell obtained from each mating. Means ± standard errors are shown; *n* ≥ 4. Statistical analysis was carried out using a Mann-Whitney *U* test, with no significant difference observed. Download FIG S4, JPG file, 1.1 MB. Copyright © 2018 Vidor et al. 2018 Vidor et al. This content is distributed under the terms of the Creative Commons Attribution 4.0 International license.

## DISCUSSION

Toxin-encoding genes are regularly associated with mobile genetic elements in the clostridia, allowing their broader dissemination by horizontal gene transfer (28). *C. perfringens*, for example, carries the majority of its toxin genes on large conjugative plasmids (38), and the BoNT toxins from *C. botulinum* are often encoded on conjugative plasmids or transducing phage (28). The other members of the LCTs are also associated with mobile or likely mobile genetic elements (24, 25, 29). Here, we showed for the first time the ability of the *C. sordellii* TcsL-encoding PaLoc to undergo horizontal gene transfer via conjugation from type strain ATCC 9714 into a distantly related nontoxigenic *C. sordellii* strain (30). We identified a novel conjugation locus on pCS1-1, *cst*, which encodes the machinery required for plasmid transfer. The *cst* locus is highly conserved among all subtypes of the pCS1 family (Fig. 1), so it is likely that all members of this family can undergo conjugative transfer. Moreover, a previous phylogenetic analysis of *C. sordellii* strains indicated that different clades acquired the pCS1 plasmids in at least three separate events (30). The demonstration that pCS1-1 can undergo conjugative transfer, and the conservation of the *cst* locus among pCS1 plasmids, identified a mechanism by which members of the distinct clades may have acquired the PaLoc. These findings have important implications for *C. sordellii* disease pathogenesis since the gene encoding the major virulence factor TcsL (13) can be transferred between strains, potentially changing nontoxigenic strains to toxigenic ones through a conjugative transfer event.

The *cst* conjugation locus has homologues on other confirmed or predicted clostridial plasmids and represents a subtype of a novel clostridial conjugation locus of considerable importance, as well as suggesting a shared evolutionary history. These include botulinum neurotoxin-encoding plasmid pCLL from *C. botulinum*, *C. perfringens* plasmid pCP13, and putative plasmids of three *C. difficile* isolates (Fig. 3; see also Fig. S2). pCLL can undergo conjugative transfer between *C. botulinum* strains, and although sequence analysis was performed on pCLL, a conjugation locus was not identified (37). The transfer frequencies of pCLL ranged from 10^−5^ to 10^−8^ transconjugants per donor cell (37), which are similar to those that were seen for pCS1-1 here (10^−5^ to 10^−7^ transconjugants per donor cell). The difference in transfer frequencies observed between the donor and different recipient isolates in both species (37) may be due to factors affecting initial cell-to-cell contact, for example, the presence of agglutination factors or receptors; however, this hypothesis has not been examined experimentally. On the basis of sequence homology between the *C. sordellii cst* locus and the *C. botulinum cst*-like locus, we propose that the *cst*-like locus on pCLL is responsible for the conjugative transfer of this plasmid, although mutagenesis and phenotypic analyses of this region are required to confirm this role.

Previous studies noted that a number of ORFs on pCLL had homology to those on *C. perfringens* plasmid pCP13 (37); however, they were not identified as conjugation-related genes. pCP13 is thought to be nonconjugative (37), probably because a pCW3-like *tcp* locus is absent (39), since the only plasmid-encoded conjugation system identified in *C. perfringens* to date has been that encoded by the *tcp* locus. However, the presence of a *cst*-like locus on pCP13 suggests that this plasmid may be conjugative, which warrants further investigation. These experiments are particularly important in the context of the clinically important pCP13 derivatives that encode the novel enterotoxin BEC, which is involved in food-borne gastroenteritis (40).

Very few plasmids have been bioinformatically characterized in *C. difficile*, and their role in evolution and pathogenesis is unknown (41). Due to the presence of a *cst*-like locus and plasmid-related genes, it may be that the contigs described here represent a novel family of conjugative plasmids in *C. difficile*. Differences were observed between the *cst*-like *oriT* in *C. difficile* and *cst* (Fig. 4A). Two *C. difficile* isolates contained a fourth inverted repeat within their predicted *oriT* genes which had inserted within inverted repeat 2 (IR2). Previous studies have shown the IR2 of Mob_MG_ relaxase-encoding plasmids to be a major factor in the specificity of relaxase-induced *oriT* nicking and plasmid mobilization (35). The major alteration in the structure of these *C. difficile oriT* sequences could mean an altered mechanism of *oriT* recognition and, if studied, could help elucidate the mechanism of Mob_MG_ relaxase *oriT* recognition. Further work will need to be conducted on these *C. difficile cst*-like loci to determine their role in conjugative transfer and, potentially, to confirm their presence on true extrachromosomal elements.

While differences were observed between the pCS1 family plasmids, including the newly characterized pCS1-5 plasmid, many putative genes were found to be conserved among the members. One example, the *srtB* gene, predicted to encode a sortase enzyme, was found upstream of the PaLoc region on all pCS1 plasmids (Fig. 1). A number of predicted surface-exposed proteins containing sortase recognition domains, including CnaB encoded directly upstream of the *cst* locus, are carried on pCS1-1 (30) (Fig. 1 and 3). We hypothesized that one or more of these cell wall-anchored proteins may behave as an adhesion factor to facilitate conjugative transfer, which has also been proposed for other Gram-positive conjugation systems, including that of pIP501 (3, 42). However, inactivation of *srtB* had little impact on the conjugative transfer of pCS1-1 (see Fig. S4 in the supplemental material), suggesting that the predicted sortase plays no role in conjugation.

Another feature that is common among the pCS1 plasmids is the presence of a putative partitioning locus containing the *parA* and *parB* genes (30) (Fig. 1). Here we showed that this region functions as a partitioning locus and that it is required for stable maintenance of pCS1-1 (Fig. 2). The conservation of this partitioning locus suggests that the other pCS1 subtypes are also likely to be highly stable; however, this idea is contrary to epidemiological findings. Although virulence studies indicate that TcsL is a major virulence factor of *C. sordellii*-mediated infection (18, 20), a low rate of *tcsL* or *tcsH* carriage of ~5% to 12% is reported for *C. sordellii* strains (30, 31, 43). Clinical reports and laboratory observations indicate that the majority of disease-causing *C. sordellii* isolates are likely to encode the LCTs but that they are quickly lost upon collection and subculture, suggesting that they may be encoded on unstable mobile genetic elements (30, 44). It has previously been hypothesized that the *C. sordellii* PaLoc may be present in different genomic locations, with the pCS1 plasmids representing stable variants of a highly unstable plasmid (30). On the basis of the data presented here, this stability may be due to the *parABS* partitioning locus. In future, multiple subcultures of clinical *C. sordellii* isolates should be avoided, and genomic DNA for sequencing should be extracted as soon as the organism is isolated from the host, which may allow potentially unstable PaLoc derivatives to be detected.

The results of this study provide a new perspective of the evolution of the LCTs among the clostridia. The clostridial LCTs are homologous between species, and the PaLoc regions flanking them are also conserved (14, 30, 45), indicating a common ancestry. The LCTs from *C. perfringens* and *C. novyi* are both associated with mobile genetic elements that are able to undergo horizontal transfer (24, 25, 29), and we have now shown that the *C. sordellii*-encoded LCT genes also have this ability. The documented transfer and integration of the *C. difficile* PaLoc appeared to result from a random high-frequency recombination (Hfr) mechanism (26), which may provide an explanation for the presence of PaLoc in previously nontoxigenic *C. difficile* clades (46). However, this hypothesis does not provide any insights into how PaLoc first arose in *C. difficile*. A recent PaLoc-focused phylogenetic analysis performed using genome sequences of diverse *C. difficile* isolates predicted that the ancestral population lacked the PaLoc and that the relationship of the PaLoc between clades is closer than for the remainder of the chromosome, suggesting a more recent acquisition of PaLoc instead of sequence variation inherited from a common ancestor (46). The sequence conservation of PaLoc from *C. difficile* and *C. sordellii* has suggested they are evolutionarily related (27, 30), which is further supported by the finding that *C. difficile* is the closest relative to *C. sordellii* (30). We speculate that an ancestral version of the *C. sordellii* pCS1 plasmids may have undergone interspecies transfer into a *C. difficile* ancestor, allowing the integration of the PaLoc onto the *C. difficile* chromosome either through random integration or via an unknown site-specific mechanism. The presence of a *cst*-like locus within *C. difficile* isolates (Fig. 3; see also Fig. S2) could support this proposition. The results of this study have broad implications for the dissemination of toxins and, potentially, antibiotic resistance genes among a diverse range of clostridial species, providing these microorganisms with a survival advantage within the infected host.

## MATERIALS AND METHODS

### Strains, plasmids, and bacterial culture.

For a full list of bacterial strains and plasmids used in this work, please refer to Table S2 andS3 in the supplemental material, respectively. Unless stated otherwise, *C. sordellii* isolates were grown in HIS broth (37 g/liter heart infusion broth [Oxoid], 5 g/liter yeast extract, 1 g/liter l-cysteine, 0.375% [wt/vol] glucose) or on HIS agar (HIS broth with 15 g/liter agar) at 37°C in an anaerobic chamber (Coy Laboratory Products, Inc.) in an atmosphere of 10% H_2_, 10% CO_2_, and 80% N_2_. *Escherichia coli* strains were grown in 2YT broth (16 g/liter tryptone, 10 g/liter yeast extract, 5 g/liter NaCl) or on 2YT agar (2YT broth with 15 g/liter agar) at 37°C. When required after sterilization of media, the following antibiotics were included at the indicated concentrations (unless otherwise specified): erythromycin (Erm; 10 μg/ml), tetracycline (Tet; 10 μg/ml), chloramphenicol (Cm; 25 μg/ml), thiamphenicol (10 μg/ml), streptomycin (Str; 200 μg/ml), rifampin (Rif; 20 μg/ml), anhydrous tetracycline (AnTet; 20 ng/ml), and d-cycloserine (DCy; 250 μg/ml).

10.1128/mBio.01761-17.6 TABLE S2  Bacterial strains used in this analysis. Download TABLE S2, PDF file, 0.5 MB. Copyright © 2018 Vidor et al. 2018 Vidor et al. This content is distributed under the terms of the Creative Commons Attribution 4.0 International license.

10.1128/mBio.01761-17.7 TABLE S3  Plasmids used in this analysis. Download TABLE S3, PDF file, 0.6 MB. Copyright © 2018 Vidor et al. 2018 Vidor et al. This content is distributed under the terms of the Creative Commons Attribution 4.0 International license.

### Molecular techniques.

*C. sordellii* genomic DNA was extracted from 5 ml of overnight culture as previously described (47), with the omission of RNase upon DNA resuspension. Plasmid DNA from *E. coli* and *C. sordellii* was extracted using a QIAprep Spin Miniprep kit (Qiagen), with the addition of 10 mg/ml lysozyme in buffer P1 followed by incubation at 37°C for 15 min for *C. sordellii*. Chemically competent *E. coli* DH5α and HB101 cells were prepared and transformed with plasmid DNA as previously described (48). PCR was performed at a final concentration of 0.5 μM of each primer, using either *Taq* (Roche) or Phusion High Fidelity (NEB) DNA polymerases. For a list of primers used for PCR in this study, please refer to Table S4. PCR products were extracted using an Ultraclean 15 DNA purification kit (Mo Bio Laboratories, Inc.). All plasmids constructed as part of this study were confirmed by restriction digestion (Roche, NE) per the manufacturer’s instructions and by agarose gel electrophoresis and Sanger sequencing analysis. Sequencing reactions were performed using Prism BigDye Terminator Mix (Applied Biosystems), and sequences were derived using an Applied Biosystems 3730S capillary sequencer. Sequences were analyzed using the Vector NTI suite (Invitrogen).

10.1128/mBio.01761-17.8 TABLE S4  Oligonucleotide primers used in PCR. (+), forward primer; (-), reverse primer. Download TABLE S4, PDF file, 0.2 MB. Copyright © 2018 Vidor et al. 2018 Vidor et al. This content is distributed under the terms of the Creative Commons Attribution 4.0 International license.

### Genome sequencing, annotation, and analysis.

*C. sordellii* genomic DNA was sequenced using either an Illumina Mi-Seq or Illumina Hi-Seq 1500 platform, with 150-bp reads produced using paired-end chemistry. Reads were quality trimmed using Nesoni clip (https://github.com/Victorian-Bioinformatics-Consortium/nesoni), followed by *de novo* assembly using SPAdes (49). For DLL5078, adapters were removed, and reads were assembled *de novo* using CLC genomics workbench version 7.0.3 (Qiagen). Plasmid sequences were closed using PCR. Autoannotation of sequences was performed using Prokka (50). Sequences not obtained as part of this study were obtained from GenBank (51), with primary accession numbers for each sequence listed in Table S2 and S3 (also see below). Sequences were further manually annotated using Artemis (Sanger) (52) on the basis of homology to entries in the Conserved Domain Database (34). Whole-plasmid comparisons and the resulting graphics were produced using the BLAST ring image generator (BRIG) tool (36). Sequence homology between conjugation loci was determined, and the resulting graphics were produced using Easyfig (53). Clustal Omega multiple-sequence alignments were conducted using EMBL-EBI tools (https://www.ebi.ac.uk/Tools/msa/clustalo/). Transmembrane helices were predicted using CBS TMHMM server 2.0 (http://www.cbs.dtu.dk/services/TMHMM).

### Mating of *C. sordellii* to *C. sordellii* pCS1-1.

Initial ATCC 9714 × R28058 matings were performed using a method for *C. perfringens* mixed-plate matings on solid media, as previously described (54, 55). Optimized matings of *C. sordellii* to *C. sordellii* were carried out using newly constructed recipient isolate DLL5153 as follows. Overnight 20-ml cultures of donor and recipient were used to inoculate fresh 20-ml broths to produce an optical density at 600 nm (OD_600_) of 0.05. For isolates containing a pRPF185-based vector, trimethoprim (Tm) was included in overnight broths to ensure the stability of the plasmid. The cultures were grown until they reached an OD_600_ of 1.2 ± 0.2 (mid-to-late exponential phase). Cultures were mixed in a donor/recipient ratio of 1:4 (with a final volume of 1.4 ml) and were then pelleted at 17,000 × *g* for 1 min. The mating pellet was resuspended in 200 μl sterile HIS diluent (3.7 g/liter heart infusion broth [Oxoid], 0.5 g/liter yeast extract) and spread on a single thick HIS plate. For complementation matings with isolates carrying a pRPF185 derivative, the HIS plate contained AnTet to switch on transcription of the complementation product. The mating plate was incubated at 37°C in an anaerobic atmosphere (AnO_2_) for ~18 h. Growth on the mating plate was removed into 3 ml of HIS diluent, followed by pipetting and brief vortex mixing to produce a homogenized culture. Serial 10-fold dilutions of the culture were carried out in HIS diluent, and duplicate 50-μl aliquots of 10^−4^-to-10^−7^ dilutions were plated onto separate halves of HIS plates containing Erm, Str, and Rif (Erm-Str-Rif) to obtain donor and recipient counts, respectively. Ten 100-μl aliquots of the undiluted reaction mixture and two aliquots of the 1-in-10 dilution were plated onto separate Erm-Str-Rif HIS plates to obtain transconjugant counts. The transfer frequencies of pCS1-1 are reported as either the number of Erm^r^ transconjugants per milliliter of mating mixture or the number of Erm^r^ transconjugants per donor cell. The detection limit for *cstD4* mutants was 1.61 × 10^−10^ to 5.56 × 10^−10^ transconjugants/donor cell, and the detection limit for *cstB4* mutants was 2.94 × 10^−10^ to 3.23 × 10^−10^ transconjugants/donor cell. Statistical analysis was performed using the Mann-Whitney *U* test.

### pCS1-1 stability assays.

Pure *C. sordellii* cultures from solid HIS media containing Erm were used to inoculate a 20-ml BHI broth (37 g/liter brain heart infusion broth [Difco], 1 g/liter sodium thioglycolate, 0.375% [wt/vol] glucose) containing Erm and grown overnight. The next morning, a 200-μl sample of this overnight culture was used to inoculate a 20-ml volume of fresh BHI broth with no selection, thereby producing a 1/100 dilution, and the fresh culture was grown throughout the day. This subculturing continued at intervals of 10 to 14 h until the original overnight culture had been subcultured a total of 12 times (~120 generations). Just prior to subculture of the original overnight culture and subcultures 4, 8, and 12, a sample was taken, serially diluted in BHI diluent (3.7 g/liter brain heart infusion broth [Difco], 0.1 g/liter sodium thioglycolate), and plated onto BHIS plates (37 g/liter brain heart infusion broth [Difco], 5 g/liter yeast extract, 15 g/liter agar, 1 g/liter l-cysteine, 0.375% [wt/vol] glucose) to obtain single colonies. For each time point and strain, 100 single colonies were patched onto BHIS plates containing Erm with no selection. The proportion of plasmid-carrying isolates was considered to be representative of the percentage of viable colonies resistant to Erm. To construct the new recipient (DLL5153), a plasmid-free isolate of *parB* mutant DLL5142 was plated onto HIS plates containing Str, and resistant isolates were selected. Str^r^ isolates were then plated onto HIS plates containing Rif, and resistant isolates were selected. The absence of pCS1-1 in DLL5153, a *C. sordellii* isolate of the ATCC 9714 background that was now Str^r^ Rif^r^, was confirmed using PCR.

### Mutagenesis.

Insertional inactivation of *C. sordellii* genes was carried out using a TargeTron system. Potential intron insertion sites were identified using the Perutka Algorithm (56) and the ClosTron intron design tool (http://clostron.com/clostron2.php). The following sites (distances from the predicted start of the coding sequence) were chosen for insertion of the group II intron: *parB* (117/118-bp sense strand), *cstD4* (1,611/1,612-bp sense strand), *cstB4* (1,392/1,393-bp sense strand), and *srtB* (621/622-bp sense strand). Intron retargeting to these sites was performed with splicing by overhang extension PCR (SOE PCR) using retargeting primers as previously described (57). The retargeted 350-bp product was cloned into clostridial TargeTron vector pDLL46 between the HindIII and BsrGI sites. The retargeted plasmids were introduced into *C. sordellii* ATCC 9714 using RP4-mediated conjugation from an *E. coli* donor containing plasmid pVS520, as previously described (20). Isolated *C. sordellii* transconjugants were inoculated into 20-ml cultures containing Tm and AnTet and grown overnight to induce production of the intron. Putative insertional inactivation mutants were selected for by plating onto HIS Erm agar and then cross patching onto HIS Tm agar. Correct mutants were Erm^r^ but Tm^s^ due to the loss of the TargeTron vector. Correct insertions of the intron and the loss of the plasmid were confirmed using PCR and Southern hybridization. Southern hybridization was carried out as previously described (58) using gene-specific (gene of interest), intron-specific (*ermB*), and vector-specific (*catP*) digoxigenin (DIG)-labeled DNA probes. All probes were labeled using random labeling PCR according to the manufacturer’s instructions (Roche). Hybridization was detected using the CDP-Star (Roche) detection system according to the manufacturer’s instructions.

### Complementation of *cstD4* and *cstB4* mutants.

The complementation strategy was based on a previously described method (59, 60). In summary, the ORFs of *cstD4* and *cstB4* were PCR amplified from ATCC 9714 genomic DNA using primer DLP634 plus primer DLP574 for *cstD4* and primer DLP635 plus primer DLP636 for *cstB4*. DLP634 and DLP635 contained a SacI restriction site, and DLP574 and DLP635 contained a BamHI restriction site. The PCR products were digested with these two enzymes and cloned into pRPF185 vector, thereby placing the complementation products under the control of a tetracycline-inducible promoter. Complementation and vector control (pRPF185) plasmids were then introduced into the mutants using RP4-mediated conjugation from an *E. coli* donor containing the pVS520 plasmid, as previously described (20).

### Data availability.

Data referred to in this article have been submitted to Genbank under accession numbers MG205641, MG205642, and MG205643 (*C. sordellii*).
