# Supplementary figures and images for: Clostridium sordellii Pathogenicity Locus Plasmid pCS1-1 Encodes a Novel Clostridial Conjugation Locus
Source: mBio. 2018 Jan 16;9(1):e01761-17. doi: 10.1128/mBio.01761-17 (PMC5770547; doi:10.1128/mBio.01761-17)

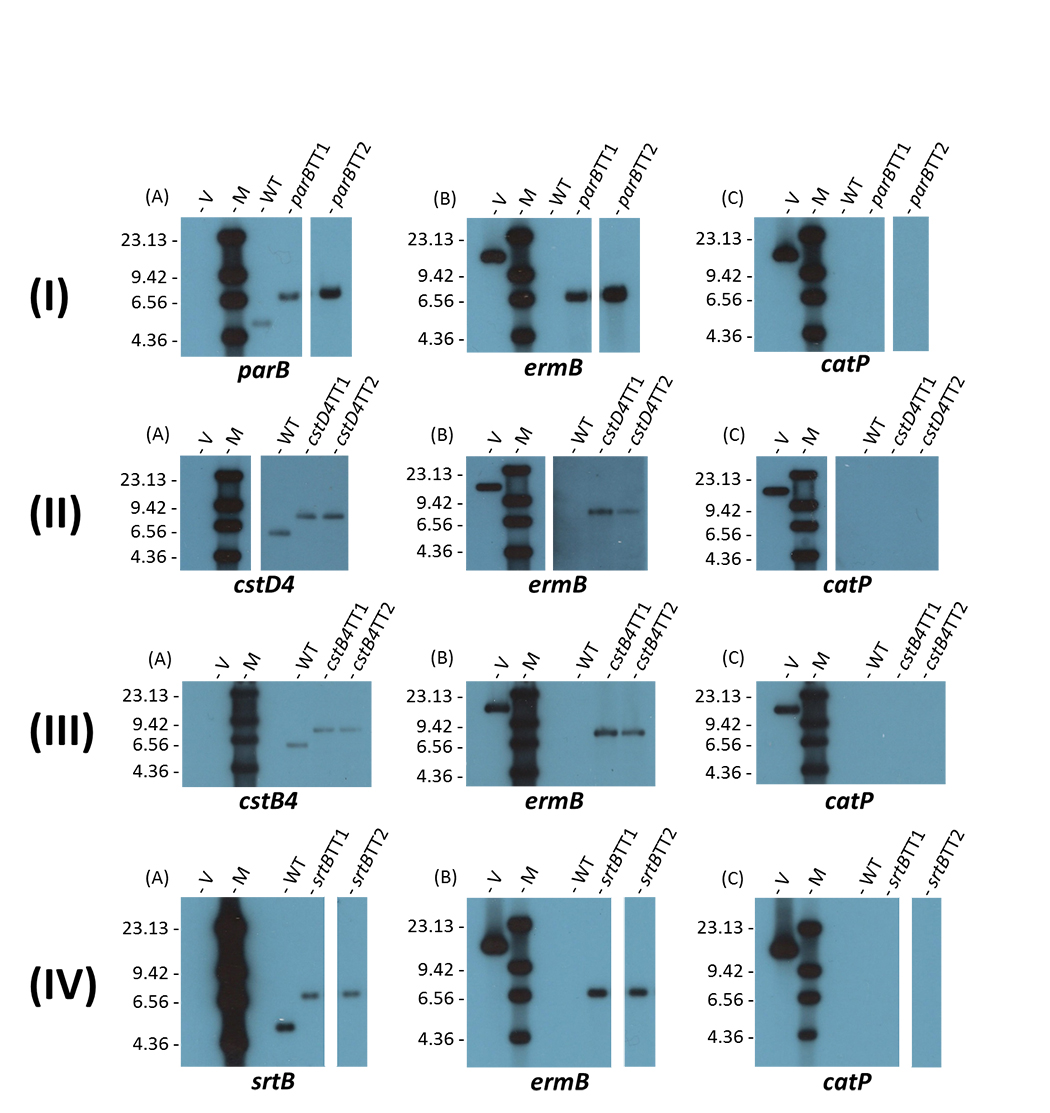

Supplement: FIG S1 [file mbo001183687sf1.jpg]

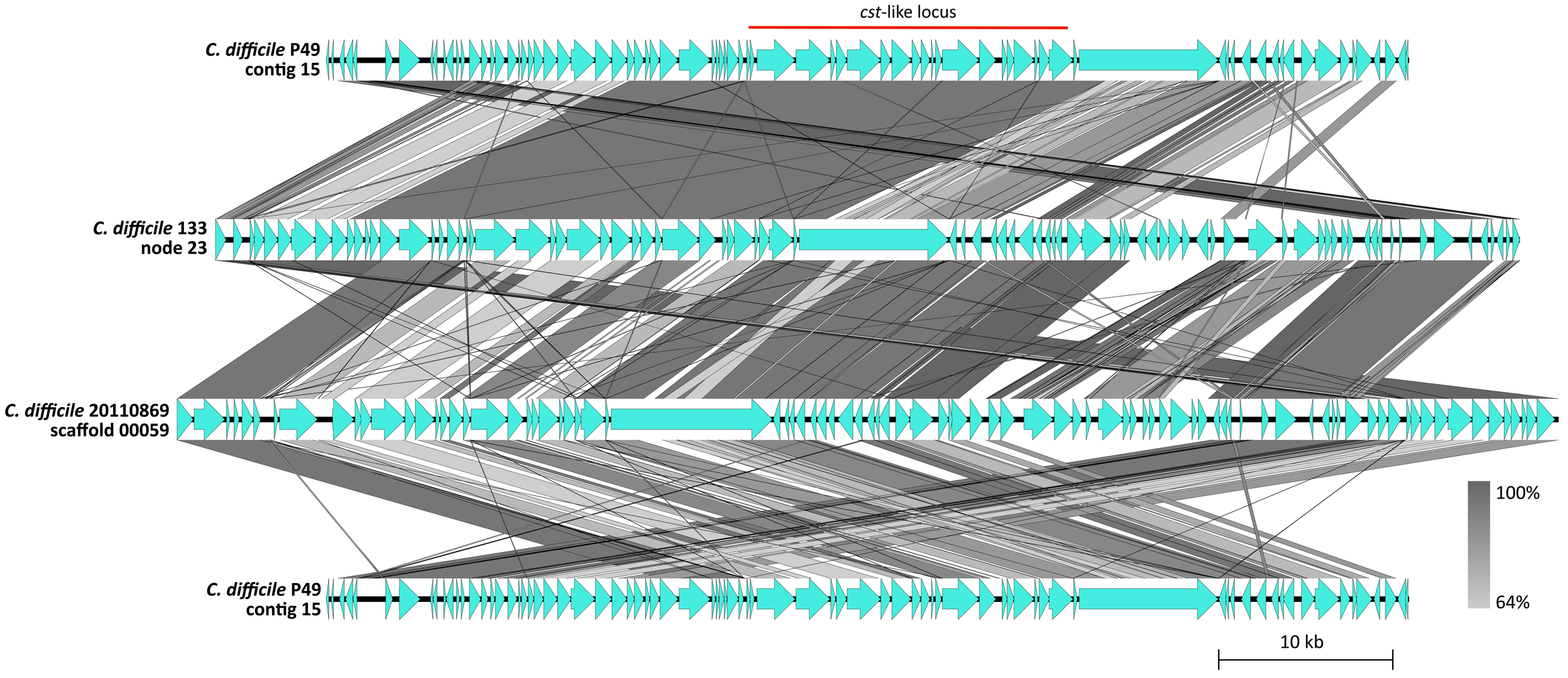

Supplement: FIG S2 [file mbo001183687sf2.jpg]

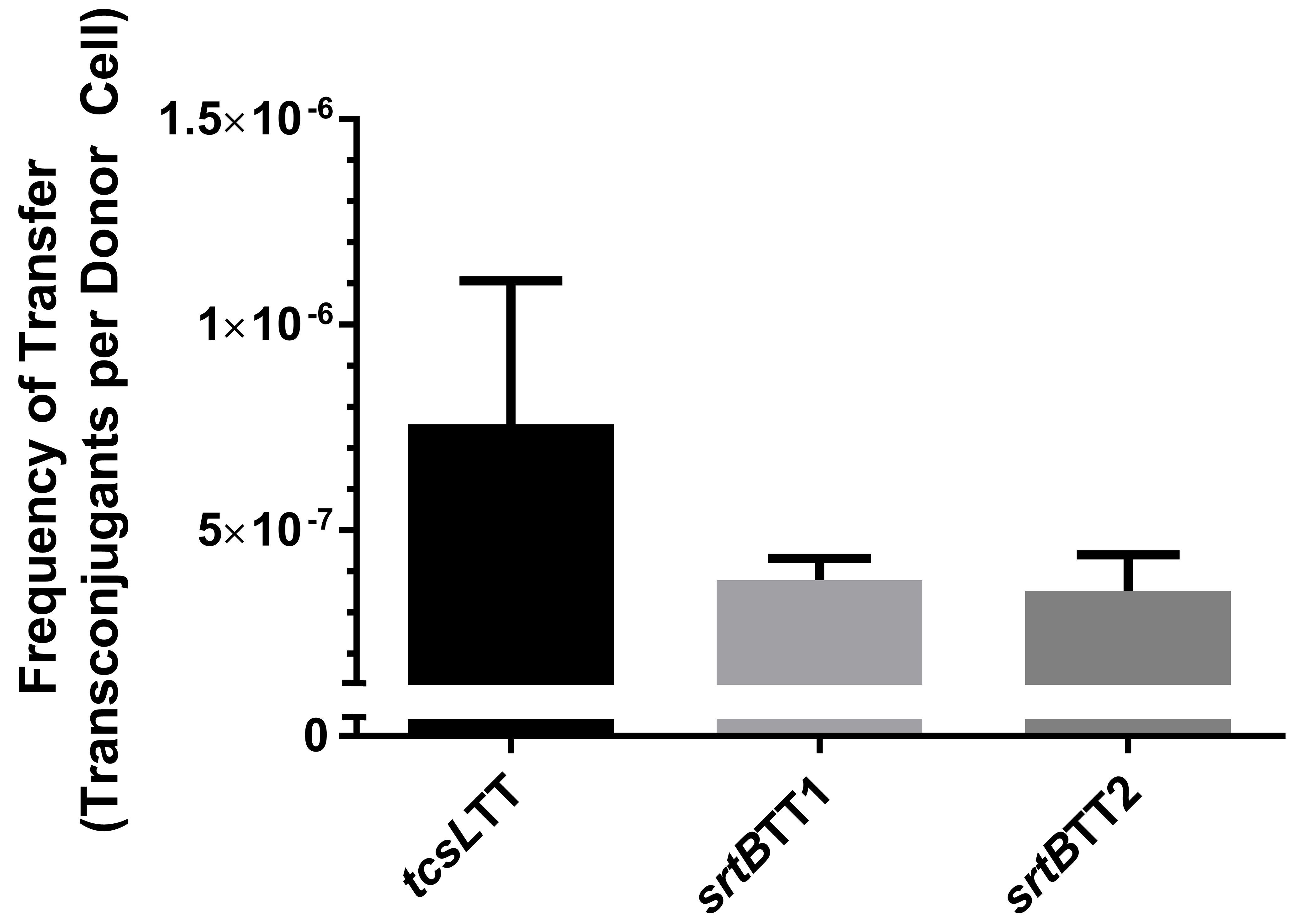

Supplement: FIG S4 [file mbo001183687sf4.jpg]
