# Supplementary material for: Clostridium sordellii Pathogenicity Locus Plasmid pCS1-1 Encodes a Novel Clostridial Conjugation Locus
Source: mBio. 2018 Jan 16;9(1):e01761-17. doi: 10.1128/mBio.01761-17 (PMC5770547; doi:10.1128/mBio.01761-17)
Supplement: TABLE S1 [file mbo001183687st1.pdf]

**Locus tag** ATCC9714PCS11_"x"	**ORF**	**Predicted function**	**pCP13 homologue (aa identity)**	**pCLL homologue (aa identity)**	***C. difficile* P49 homologue (aa identity)**	**pCP13 vs. pCLL (aa identity)**	**pCP13 vs. CdP49 (aa identity)**	**pCLL vs. CdP49 (aa identity)**
00231	*cnaB*	Surface protein, putative adhesion factor	*pcp57/cnaB* (43.0%)	-	-	-	-	-
00241	*241*	Unknown	-	-	-	-	-	-
00251	*251*	Unknown	-	-	-	-	-	-
00261	*topA*	Type I Topoisomerase	*pcp47/topA* (50.9%)	-	QUA_0964 (58.8%)	-	-	-
00271	*271*	Unknown	-	-	-	-	-	-
00281	*281*	Transmembrane protein	-	-	-	-	-	-
00291	*cstA*	Membrane protein	*pcp53* (40.8%)	*cll4* (35.5%)	QUA_0952 (35.5%)	68.0%	56.4%	55.1%
00301	*cstD4*	VirD4-like ATPase (Coupling Protein)	*pcp51* (49.3%)	*cll5* (51.9%)	QUA_0953 (55.5%)	55.4%	49.2%	51.8%
00311	*cstB6*	VirB6-like membrane channel protein	*pcp50* (35.5%)	*cll6* (28.7%)	QUA_0954 (32.2%)	37.4%	29.1%	28.5%
00321	*cstE*	Unknown	*pcp49* (38.0%)	*cll7* (34.8%)	QUA_0955 (52.7%)	52.2%	46.7%	40.5%
00331	*cstF*	Unknown	*pcp48* (41.5%)	*cll8* (38.6%)	QUA_0956 (45.0%)	53.1%	41.6%	45.9%
00341	*cstB4*	VirB4-like ATPase	*pcp46* (51.9%)	*cll9* (52.8%)	QUA_0957 (53.3%)	63.9%	48.9%	50.9%
00351	*cstH*	Phage protein	-	-	-	-	-	-
00361	*cstI*	Unknown	-	-	QUA_0958 (27.2%)	-	-	-
00371	*cstB1*	Muramidase	*pcp44* (57.7%)	*cll11* (56.9%)	QUA_0959 (42.6%)	59.3%	45.7%	42.3%
00381	*cstK*	Single stranded DNA binding protein	-	*cll19* (30.4%)	QUA_0963 (36.0%)	-	-	27.0%
00391	*cstL*	Unknown	-	-	QUA_0965 (31.8%)	-	-	-
00401	*cstM*	CopG family protein	*pcp41* (28.6%)	*cll13* (40.3%)	QUA_0966 (27.3%)	48.3%	38.1%	39.8%

00411	*cstN*	CopG family protein	*pcp41* (40.2%)	*cll13* (41.9%)	QUA_0966 (39.3%)	48.3%	38.1%	39.8%
00421	*cstO*	Unknown	-	-	-	-	-	-
00431	*cstD2*	Relaxase	*pcp39* (43.5%)	*cll15* (44.9%)	QUA_0968 (50.9%)	53.1%	45.5%	47.1%
00441	*cstQ*	Unknown	-	-	QUA_0969 (32.3%)	-	-	-
00451	*cstR*	Unknown	*pcp36* (43.5%)	-	QUA_0971 (44.0%)	-	40.6%	-
