# Supplementary material for: Clostridium sordellii Pathogenicity Locus Plasmid pCS1-1 Encodes a Novel Clostridial Conjugation Locus
Source: mBio. 2018 Jan 16;9(1):e01761-17. doi: 10.1128/mBio.01761-17 (PMC5770547; doi:10.1128/mBio.01761-17)
Supplement: TABLE S2 [file mbo001183687st2.pdf]

Strain	Characteristics	Reference	Accession number
Clostridium sordellii			
ATCC9714	Reference, cause of oedema, contains pCS1-1	(1), (2)	LN679998
R28058	Isolated from infected crushed hand, Tet^R	(2)	CEKZ00000000
7543-A	Unknown origin, pCS1-6	(3)	N/A
7508-A	Unknown origin, pCS1-7	(3)	N/A
S0804018	Equine intestinal disease, pCS1-5	This study	N/A
DLL5002 [*tcsL*TT]	ATCC9714(pDLL230), Erm^R	(4)	N/A
DLL5078	DLL5002xR28058 transconjugant, R28058(pDLL230), Tet^R, Erm^R	This study	N/A
DLL5138 [*parB*TT1]	ATCC9714(pCS1-1Ω*parB*::TT), independent mutant 1, Erm^R	This study	N/A
DLL5142 [*parB*TT2]	ATCC9714(pCS1-1Ω*parB*::TT), independent mutant 2, Erm^R	This study	N/A
DLL5153	pCS1-1 free DLL5142, Str^R, Rif^R	This study	N/A
DLL5132 [*srtB*TT1]	ATCC9714(pCS1-1Ω*srtB*::TT), independent mutant 1, Erm^R	This study	N/A
DLL5134 [*srtB*TT2]	ATCC9714(pCS1-1Ω*srtB*::TT), independent mutant 2, Erm^R	This study	N/A
DLL5181 [*cstD4*TT1]	ATCC9714(pCS1-1Ω*cstD4*::TT), independent mutant 1, Erm^R	This study	N/A
DLL5183 [*cstD4*TT2]	ATCC9714(pCS1-1Ω*cstD4*::TT), independent mutant 2, Erm^R	This study	N/A
DLL5188 [*cstB4*TT1]	ATCC9714(pCS1-1Ω*cstB4*::TT), independent mutant 1, Erm^R	This study	N/A
DLL5190 [*cstB4*TT2]	ATCC9714(pCS1-1Ω*cstB4*::TT), independent mutant 2, Erm^R	This study	N/A
DLL5216 [*cstD4*TT(*cstD4*$^+$)]	DLL5181(pDLL183), Erm^R, Tm^R	This study	N/A
DLL5217 [*cstD4*TT(V)]	DLL5181(pRPF185), Erm^R, Tm^R	This study	N/A
DLL5232 [*cstB4*TT(*cstB4*$^+$)]	DLL5188(pDLL212), Erm^R, Tm^R	This study	N/A
DLL5230 [*cstB4*TT(V)]	DLL5188(pRPF185), Erm^R, Tm^R	This study	N/A
C. difficile			
P49	Unknown origin, contig 15 analysed	N/A	AVMN01000016
133	Human, node 23 analysed	N/A	NZ_MBGB01000023
20110869	Unknown origin, scaffold 00059 analysed	N/A	NZ_MTVT01000059

1. **Hall IC, Scott, J.P.** 1927. *Bacillus sordellii*, a Cause of Malignant Edema in Man. J Infect Dis **41:**329-335.
2. **Couchman EC, Browne HP, Dunn M, Lawley TD, Songer JG, Hall V, Petrovska L, Vidor C, Awad M, Lyras D, Fairweather NF.** 2015. *Clostridium sordellii* genome analysis reveals plasmid localized toxin genes encoded within pathogenicity loci. BMC Genomics **16:**392.
3. **Thiele TL, Stuber TP, Hauer PJ.** 2013. Detection of *Clostridium sordellii* strains expressing hemorrhagic toxin (TcsH) and implications for diagnostics and regulation of veterinary vaccines. Vaccine **31:**5082-5087.
4. **Carter GP, Awad MM, Hao Y, Thelen T, Bergin IL, Howarth PM, Seemann T, Rood JI, Aronoff DM, Lyras D.** 2011. TcsL is an essential virulence factor in *Clostridium sordellii* ATCC 9714. Infect Immun **79:**1025-1032.
