# Supplementary material for: Clostridium sordellii Pathogenicity Locus Plasmid pCS1-1 Encodes a Novel Clostridial Conjugation Locus
Source: mBio. 2018 Jan 16;9(1):e01761-17. doi: 10.1128/mBio.01761-17 (PMC5770547; doi:10.1128/mBio.01761-17)
Supplement: TABLE S3 [file mbo001183687st3.pdf]

Plasmid	Characteristics	Reference	Accession number
pCS1-1	*C. sordellii* ATCC9714 pCS1 plasmid, *tcsL*$^+$, *tcsH*$^-$, *tcs*$^+$	(1)	LN679999
pCS1-2	*C. sordellii* JGS6364 pCS1 plasmid, *tcsL*$^+$, *tcsH*$^-$, *tcs*$^+$	(1)	LN681232
pCS1-3	*C. sordellii* JGS6382 pCS1 plasmid, *tcsL*$^+$, *tcsH*$^+$, *tcs*$^+$	(1)	LN681235
pCS1-4	*C. sordellii* UMC2 pCS1 plasmid, *tcsL*$^-$, *tcsH*$^-$, *tcs*$^+$	(1)	LN681233
pCS1-5	*C. sordellii* S0804018 pCS1 plasmid, *tcsL*$^+$, *tcsH*$^-$, *tcs*$^+$	This study	MG205643
pCS1-6	*C. sordellii* 7543-A pCS1 plasmid, *tcsL*$^+$, *tcsH*$^+$, *tcs*$^+$	This study	MG205642
pCS1-7	*C. sordellii* 7508-A pCS1 plasmid, *tcsL*$^+$, *tcsH*$^+$, *tcs*$^+$	This study	MG205641
pDLL230	pCS1-1Ω*tcsL*::TT	(2)	N/A
pIP501	Broad host range conjugative plasmid, contains Tra locus	(3)	AJ505823, L39769
pCW3	*C. perfringens* conjugative tetracycline resistance plasmid	(4)	DQ366035
pCP13	*C. perfringens*, encodes beta2 toxin	(5)	AP003515
pCLL	*C. botulinum* conjugative neurotoxin plasmid	(6)	CP001057
pVS520	Tra$^+$, Mob$^+$, RP4 derivative, Tet^R	(7)	N/A
pDLL46	pMTL9361 derivative with BsrGI and HindIII sites removed from *rep*, contains RP4 and Tn*916* oriTs and *lacZ*α within retargeting region for blue white screening, Cm^R/Tm^R	This study	N/A
pDLL116	pDLL46 retargeted to 621/622s site of *srtB*, Cm^R/Tm^R	This study	N/A
pDLL120	pDLL46 retargeted to 117/118s site of *parB*, Cm^R/Tm^R	This study	N/A
pDLL143	pDLL46 retargeted to 1611/1612s site of *cstD4*, Cm^R/Tm^R	This study	N/A
pDLL147	pDLL46 retargeted to 1392/1393s site of *cstB4*, Cm^R/Tm^R	This study	N/A
pRPF185	Clostridial tetracycline inducible expression vector, Cm^R/Tm^R	(8)	N/A
pDLL183	pRPF185 carrying *cstD4* with its predicted RBS under a tetracycline inducible promoter, Cm^R/Tm^R	This study	N/A
pDLL212	pRPF185 carrying *cstB4* with its predicted RBS under a tetracycline inducible promoter, Cm^R/Tm^R	This study	N/A
pWBG745	*S. aureus* pWBG749-family plasmid	(9)	GQ900389

1. **Couchman EC, Browne HP, Dunn M, Lawley TD, Songer JG, Hall V, Petrovska L, Vidor C, Awad M, Lyras D, Fairweather NF.** 2015. *Clostridium sordellii* genome analysis reveals plasmid localized toxin genes encoded within pathogenicity loci. BMC Genomics **16:**392.
2. **Carter GP, Awad MM, Hao Y, Thelen T, Bergin IL, Howarth PM, Seemann T, Rood JI, Aronoff DM, Lyras D.** 2011. TcsL is an essential virulence factor in *Clostridium sordellii* ATCC 9714. Infect Immun **79:**1025-1032.
3. **Thompson JK, Collins MA.** 2003. Completed sequence of plasmid pIP501 and origin of spontaneous deletion derivatives. Plasmid **50:**28-35.
4. **Rood JI, Maher EA, Somers EB, Campos E, Duncan CL.** 1978. Isolation and Characterization of Multiply Antibiotic-Resistant *Clostridium perfringens* Strains from Porcine Feces. Antimicrob Agents Chemother **13:**871-880.
5. **Shimizu T, Ohtani K, Hirakawa H, Ohshima K, Yamashita A, Shiba T, Ogasawara N, Hattori M, Kuhara S, Hayashi H.** 2002. Complete genome sequence of *Clostridium perfringens*, an anaerobic flesh-eater. Proceedings of the National Academy of Sciences **99:**996-1001.
6. **Marshall KM, Bradshaw M, Johnson EA.** 2010. Conjugative Botulinum Neurotoxin-Encoding Plasmids in *Clostridium botulinum*. PLoS ONE **5:**e11087.
7. **Palombo EA, Yusoff K, Stanisich VA, Krishnapillai V, Willetts NS.** 1989. Cloning and genetic analysis of tra cistrons of the Tra 2/Tra 3 region of plasmid RP1. Plasmid **22:**59-69.
8. **Fagan RP, Fairweather NF.** 2011. *Clostridium difficile* has two parallel and essential Sec secretion systems. J Biol Chem **286:**27483-27493.
9. **O'Brien FG, Ramsay JP, Monecke S, Coombs GW, Robinson OJ, Htet Z, Alshaikh FA, Grubb WB.** 2015. *Staphylococcus aureus* plasmids without mobilization genes are mobilized by a novel conjugative plasmid from community isolates. J Antimicrob Chemother **70:**649-652.
