# Supplementary material for: Clostridium sordellii Pathogenicity Locus Plasmid pCS1-1 Encodes a Novel Clostridial Conjugation Locus
Source: mBio. 2018 Jan 16;9(1):e01761-17. doi: 10.1128/mBio.01761-17 (PMC5770547; doi:10.1128/mBio.01761-17)
Supplement: TABLE S4 [file mbo001183687st4.pdf]

Primer	Sequence (5’-3’)	Use
DLP104	GGTGGTTGATAGAGTTGGAGGTATTG	Internal *tetA*(P) (+) for screening transconjugants
DLP105	TTGCTTCCTGCATCATATACTTCTTG	Internal *tetB*(P) (-), screening transconjugants
DLP285	CTAATTTGAATCCTATATCAGGAGCGG	Internal ATCC9714PCS11_00781 (+), screening transconjugants
DLP286	TCCAACACTTAATTCATCTCCAGTAAGTG	Internal ATCC9714PCS11_00781 (-), screening transconjugants
DLP287	AGATGAAGCGATGAAGATATACTCACAAG	Internal ATCC9714PCS11_00261[*topA*] (+), screening transconjugants
DLP288	CCGTATATGTTCCTGATCCATCTTTC	Internal ATCC9714PCS11_00261[*topA*] (-), screening transconjugants
DLP289	TTAATTGGAGTTAGTGTAGGGTCGCC	Internal ATCC9714PCS11_00521 (+), screening transconjugants
DLP290	CCCAACGAATTGCAGCTCTACTAC	Internal ATCC9714PCS11_00521 (-), screening transconjugants
DLP304	CAAGAACGATTTATGTGTCTCCTACTGG	Internal R28058_18131 (+), screening transconjugants
DLP306	CATCATGCTTAACTCCCATACCTTCC	Internal R28058_18131 (-), screening transconjugants
DLP377	AACTTTCAACTCTTGAGGCAAAGG	Internal *parB* (+), screening of mutants, generation of probe
DLP378	CCTACCTGTGTAGGACTCAGTCCAG	Internal *parB* (-), screening of mutants, generation of probe
DLP383	AAAAAAGCTTATAATTATCCTTAGATAGC GACAAGGTGCGCCCAGATAGGGTG	*srtB* TargeTron IBS (+)
DLP384	CAGATTGTACAAATGTGGTGATAACAGAT AAGTCGACAAGATTAACTTACCTTTCTTTG T	*srtB* TargeTron EBS1d (-)
DLP385	TGAACGCAAGTTTCTAATTTCGATTCTATC TCGATAGAGGAAAGTGTCT	*srtB* TargeTron EBS2 (+)
DLP389	AAAAAAGCTTATAATTATCCTTAGATTTC GCTGAAGTGCGCCCAGATAGGGTG	*parB* TargeTron IBS (+)
DLP390	CAGATTGTACAAATGTGGTGATAACAGAT AAGTCGCTGAAGATAACTTACCTTTCTTTG T	*parB* TargeTron EBS1d (-)
DLP391	TGAACGCAAGTTTCTAATTTCGATTAAATC TCGATAGAGGAAAGTGTCT	*parB* TargeTron EBS2 (+)
DLP398	TGGAAGCATTTTTATGGATTATCGAG	Internal *srtB* (+), screening of mutants, generation of probe
DLP399	ATGAACTACAGTTCTTGCATTATCAAATT	Internal *srtB* (-), screening of mutants, generation of probe
DLP474	AAATCTAATTCCTCCAATACAGATTCAAA	Internal *cstD4* (+), screening of mutants, generation of probe
DLP475	ATCTAATTCCACGACCTAGACAAACTG	Internal *cstD4* (-), screening of mutants, generation of probe
DLP476	AAAAAAGCTTATAATTATCCTTAGAAGTC ACATCAGTGCGCCCAGATAGGGTG	*cstD4* TargeTron IBS (+)
DLP477	CAGATTGTACAAATGTGGTGATAACAGAT AAGTCACATCAGATAACTTACCTTTCTTTG T	*cstD4*TargeTron EBS1d (-)
DLP478	TGAACGCAAGTTTCTAATTTCGATTACTTC TCGATAGAGGAAAGTGTCT	*cstD4* TargeTron EBS2 (+)
DLP479	ATCTACTGAATATGCACCAGATAAATATC C	Internal *cstB4* (+), screening of mutants, generation of probe

DLP480	GCCTCTCTTTCAAAGCTAGTTAAATAGTCA	Internal *cstB4* (-), screening of mutants, generation of probe
DLP481	AAAAAAGCTTATAATTATCCTTAATTTTCAACGCTGTGCGCCCAGATAGGGTG	*cstB4* TargeTron IBS (+)
DLP482	CAGATTGTACAAATGTGGTGATAACAGATAAGTCAACGCTCATAACTTACCTTTCTTTGT	*cstB4* TargeTron EBS1d (-)
DLP483	TGAACGCAAGTTTCTAATTTCGGTTAAAATCCGATAGAGGAAAGTGTCT	*cstB4* TargeTron EBS2 (+)
DLP634	TTTTTTGAGCTCTTTTTTAAAGAAGGTGATTTTTATTGGATTTATTA	Amplify *cstD4* and predicted RBS (+), introduces SacI site
DLP574	CGGGATCCCGTTGATACTAAAGCCCATAAAATCCATC	Amplify *cstD4* and predicted RBS (-), introduces BamHI site
DLP635	AAAAAAGAGCTCAAAAAATTGAAAGGATGATAATAACTATGATAAAAG	Amplify *cstB4* and predicted RBS (+), introduces SacI site
DLP636	CGGGATCCCGCCGTAGGAATTTGAGGTTGTATGG	Amplify *cstB4* and predicted RBS (-), introduces BamHI site
DLP686	CTCTTCTTCAACATTATCTATGACAGAGTC	Close and sequence pCS1-3 plasmids from 7543-A and 7508-A (-)
DLP687	TGAAGGGAGGAATTACAATGAGTGAG	Close and sequence pCS1-3 plasmids from 7543-A and 7508-A (+)
DLP688	CCTAAATATCCTAGAACTCCCATATCACC	Close and sequence pCS1-5 plasmid from S0804018 (-)
DLP689	AGGAACTCTATTTAATCCAATGGCTG	Close and sequence pCS1-5 plasmid from S0804018 (+)
JRP4324	GGTAAATGGATAAATAAAGAAGAAAG	Internal *tcsL* (+), screening of transconjugants
JRP4325	GATATATGAGTAGCATATTCAGAG	Internal *tcsL* (-), screening of transconjugants
JRP3590	AATAAGTAAACAGGTAACGTCT	Internal *ermB* (+), screening of transconjugants and mutants, generation of probe
JRP4555	GTTTACTTTGGCGTGTTTCATTGC	Internal *ermB* (-), screening of transconjugants and mutants, generation of probe
JRP4589	TTACAGTTCAAAACCCAACCTATGG	Internal *sdl* (+), screening of transconjugants
JRP4590	TGCAGCTTGTACATCTTTGCTCTTA	Internal *sdl* (-), screening of transconjugants
JRP4330	CTCAGTACTGAGAGGGAACTTAGATGGTAT	Internal *catP* (+), screening of mutants, generation of probe
JRP4331	CCGGGATCCTTAGGGTAACAAAAAACACC	Internal *catP* (-), screening of mutants, generation of probe
JRP3867	CGAAATTAGAAACTTGCGTTCAGTAAAC	TargeTron EBS universal
